# Supplementary material for: Nutrient composition of Chenopodium formosanum Koidz. bran: Fractionation and bioactivity of its soluble active polysaccharides
Source: PeerJ. 2022 May 25;10:e13459. doi: 10.7717/peerj.13459 (PMC9147384; doi:10.7717/peerj.13459)

Fig. S1. Gel filtration chromatography

Fig. S1a)   fraction CF-1

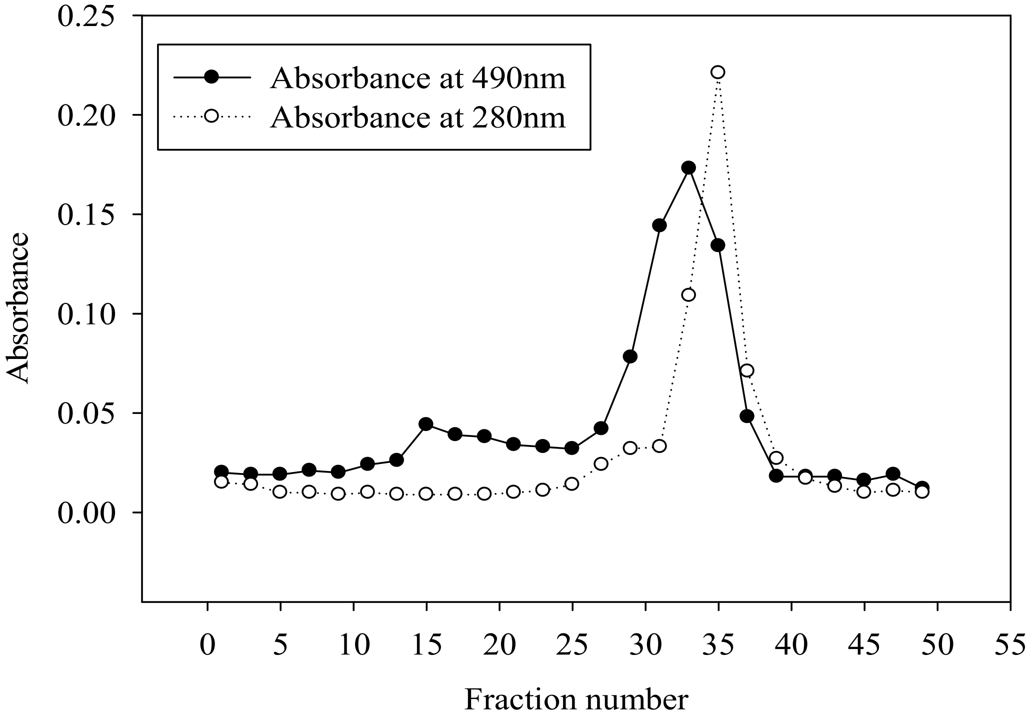

Fig. S1b)   fraction CF-2

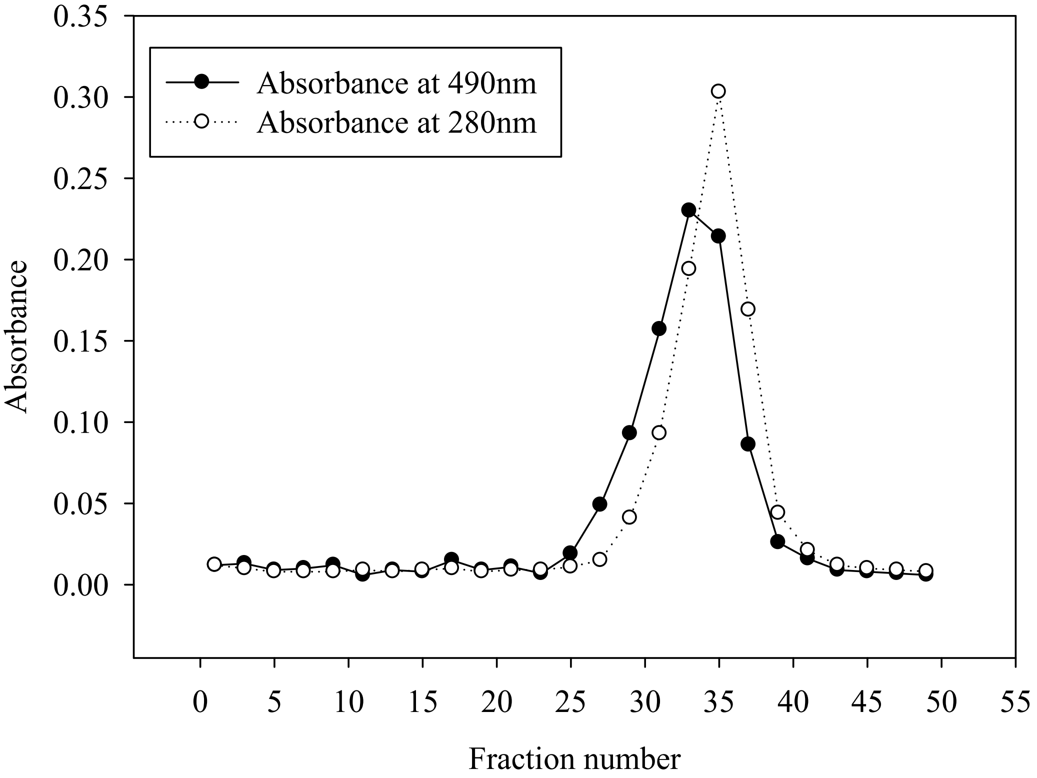

Fig. S1c) fraction CF-3

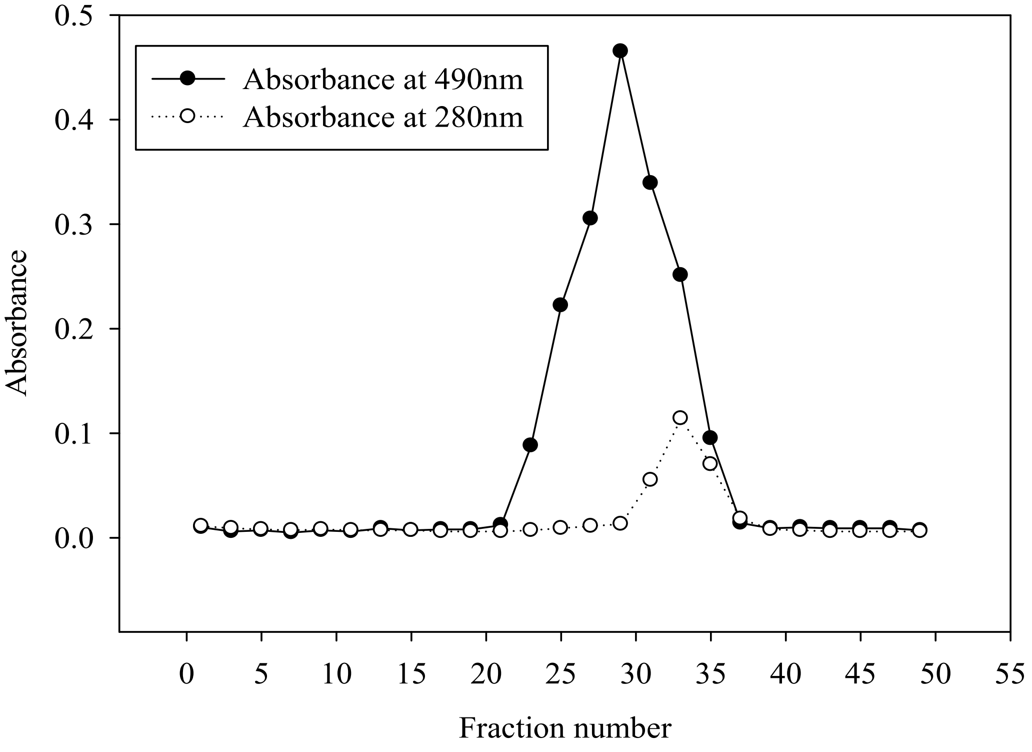

Fig. S1d) **fraction CF-4**

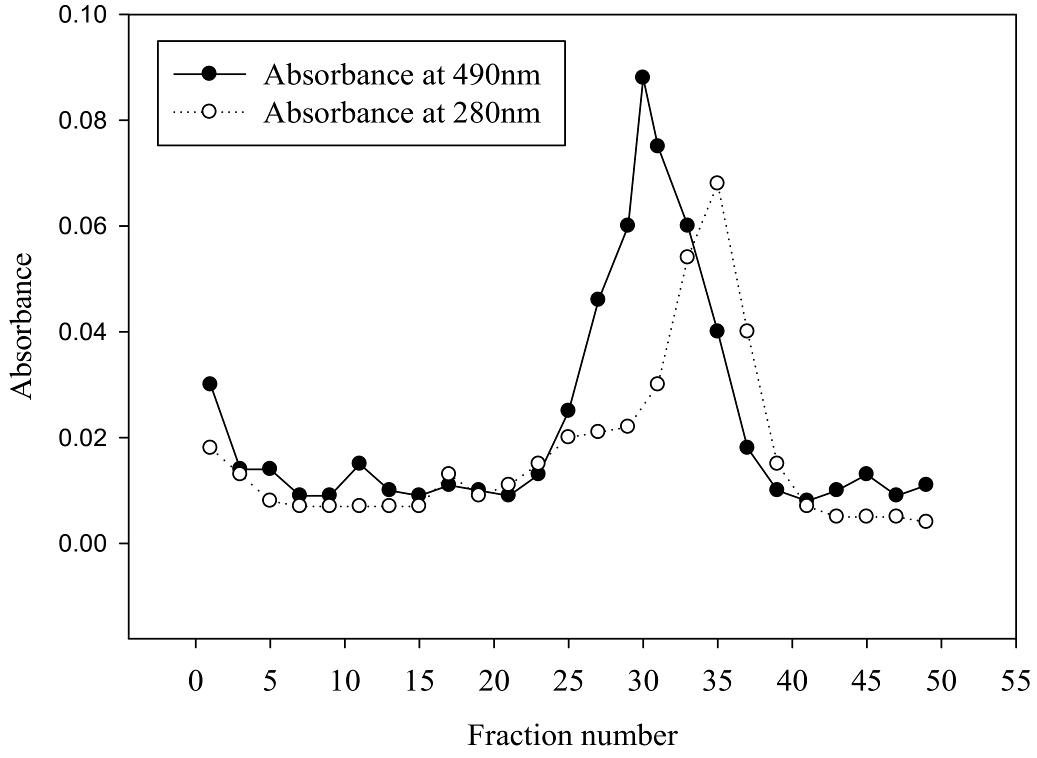

Supplement: Supplemental Information 1 — (A) CF-1: the 3-fold ethanol precipitate from the hot water extracts. (B) CF-2: the isoelectric precipitate from the 2%-NaOH extracts. (C) CF-3: the 3-fold ethanol precipitate from the 2%-NaOH extracts post isoelectric precipitation. (D) CF-4: the 3-fold ethanol precipitate from the 10%-KOH extracts post isoelectric precipitation. [file peerj-10-13459-s001.pdf]
