# Supplementary material for: Nutrient composition of Chenopodium formosanum Koidz. bran: Fractionation and bioactivity of its soluble active polysaccharides
Source: PeerJ. 2022 May 25;10:e13459. doi: 10.7717/peerj.13459 (PMC9147384; doi:10.7717/peerj.13459)
Supplement: Supplemental Information 2 — (A) CF-1: the 3-fold ethanol precipitate from the hot water extracts. (B) CF-2: the isoelectric precipitate from the 2%-NaOH extracts. (C) CF-3: the 3-fold ethanol precipitate from the 2%-NaOH extracts post isoelectric precipitation. (D) CF-4: the 3-fold ethanol precipitate from the 10%-KOH extracts post isoelectric precipitation. [file peerj-10-13459-s002.pdf]

Fig. S2. HPLC

Fig. S2a) CF-1 HPLC

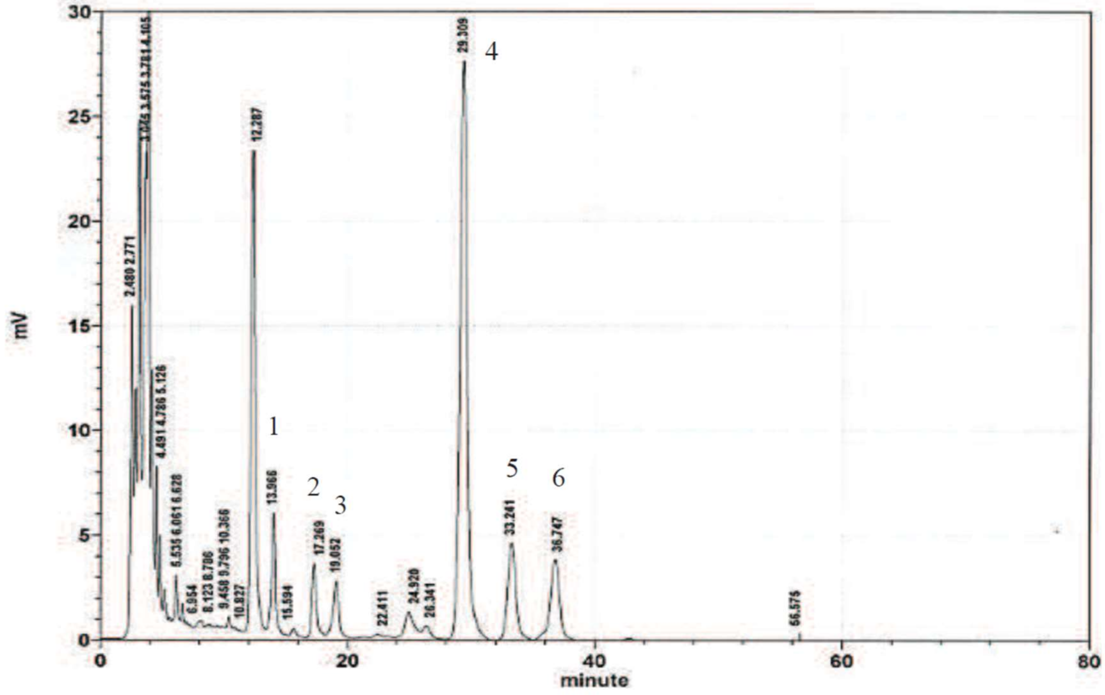

Peak assignment: 1: Mannose; 2: Glucuronic acid; 3: Rhamnose; 4: Glucose; 5: Galactose; 6: Xylose

Fig. S2b) CF-2 HPLC

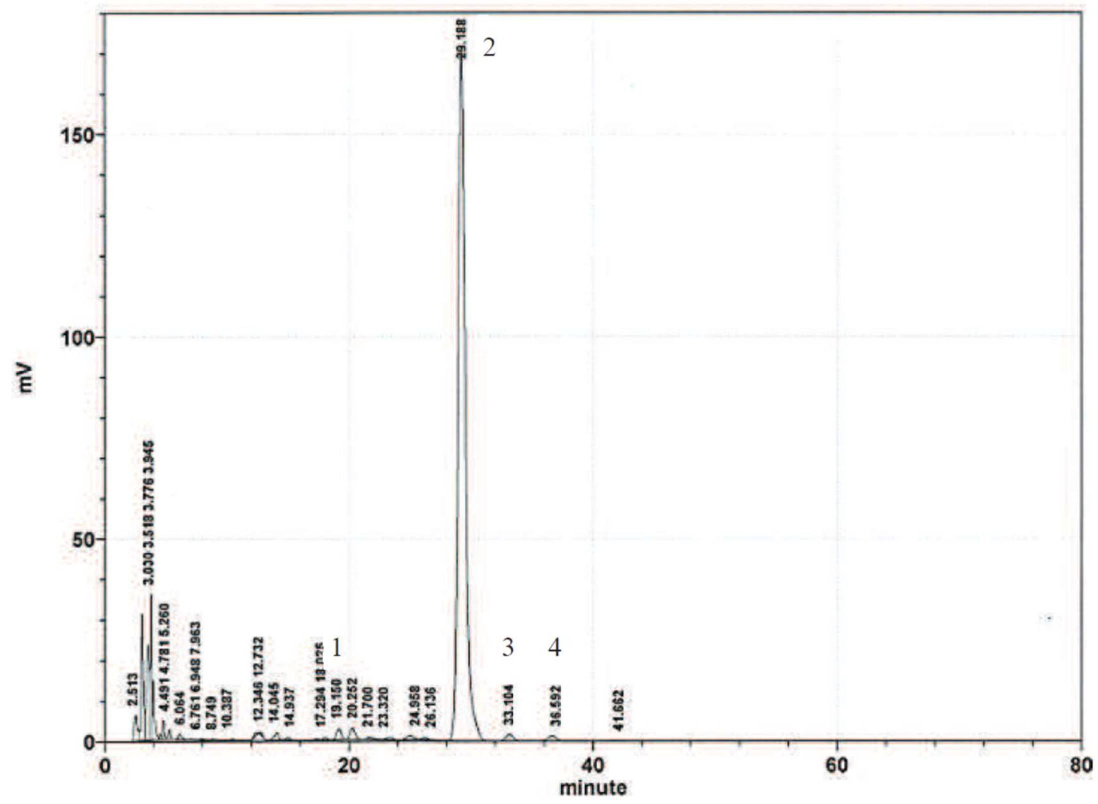

Peak assignment: 1: Rhamnose; 2: Glucos ;23: Galactose; 4: Xylose.

Fig. S2c) CF-3 HPLC

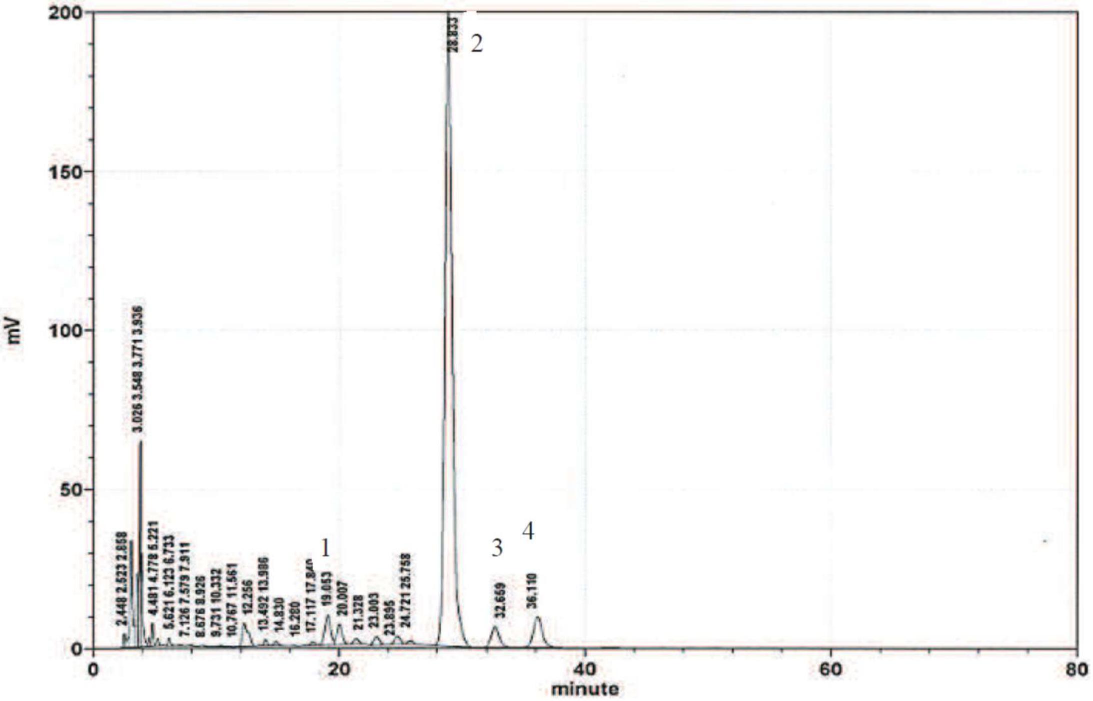

Peak assignment: 1: Rhamnose; 2: Glucose; 3: Galactose; 4: Xylose.

Fig. S2d) **CF-4** **HPLC**

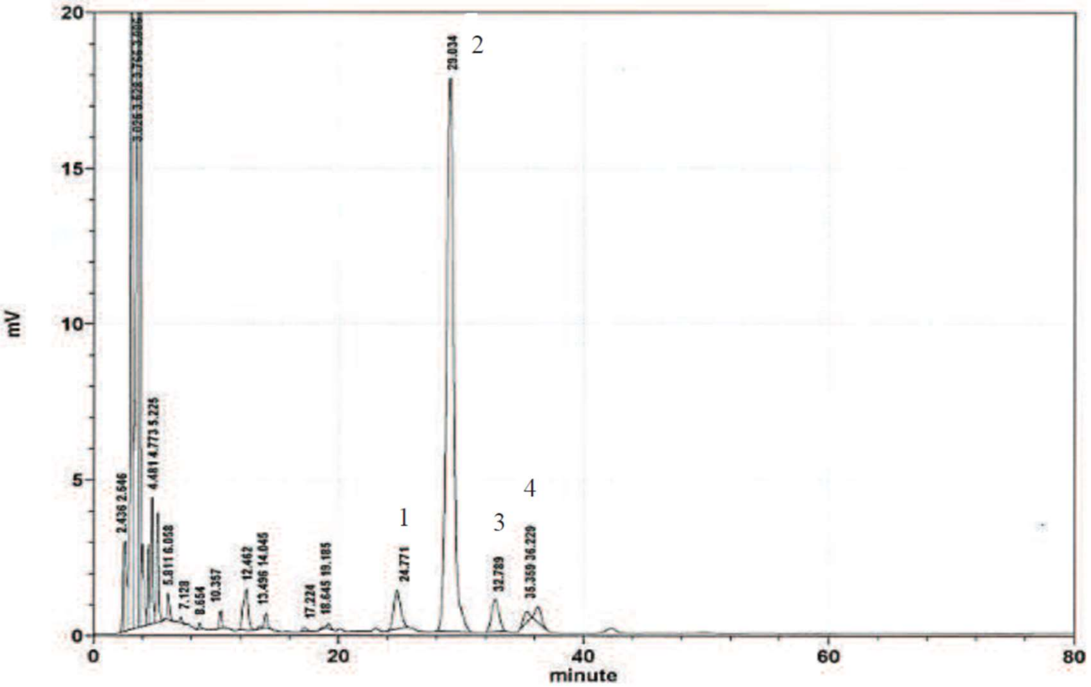

Peak assignment: 1: Galacturonic acid; 2: Glucose; 3: Galactose; 4: Xylose.
