# Supplementary material for: Nutrient composition of Chenopodium formosanum Koidz. bran: Fractionation and bioactivity of its soluble active polysaccharides
Source: PeerJ. 2022 May 25;10:e13459. doi: 10.7717/peerj.13459 (PMC9147384; doi:10.7717/peerj.13459)
Supplement: Supplemental Information 3 — (A) CF-1: the 3-fold ethanol precipitate from the hot water extracts. (B) CF-2: the isoelectric precipitate from the 2%-NaOH extracts. (C) CF-3: the 3-fold ethanol precipitate from the 2%-NaOH extracts post isoelectric precipitation. (D) CF-4: the 3-fold ethanol precipitate from the 10%-KOH extracts post isoelectric precipitation. [file peerj-10-13459-s003.pdf]

Fig. S3 GC

Fig. S3a) GC CF-1

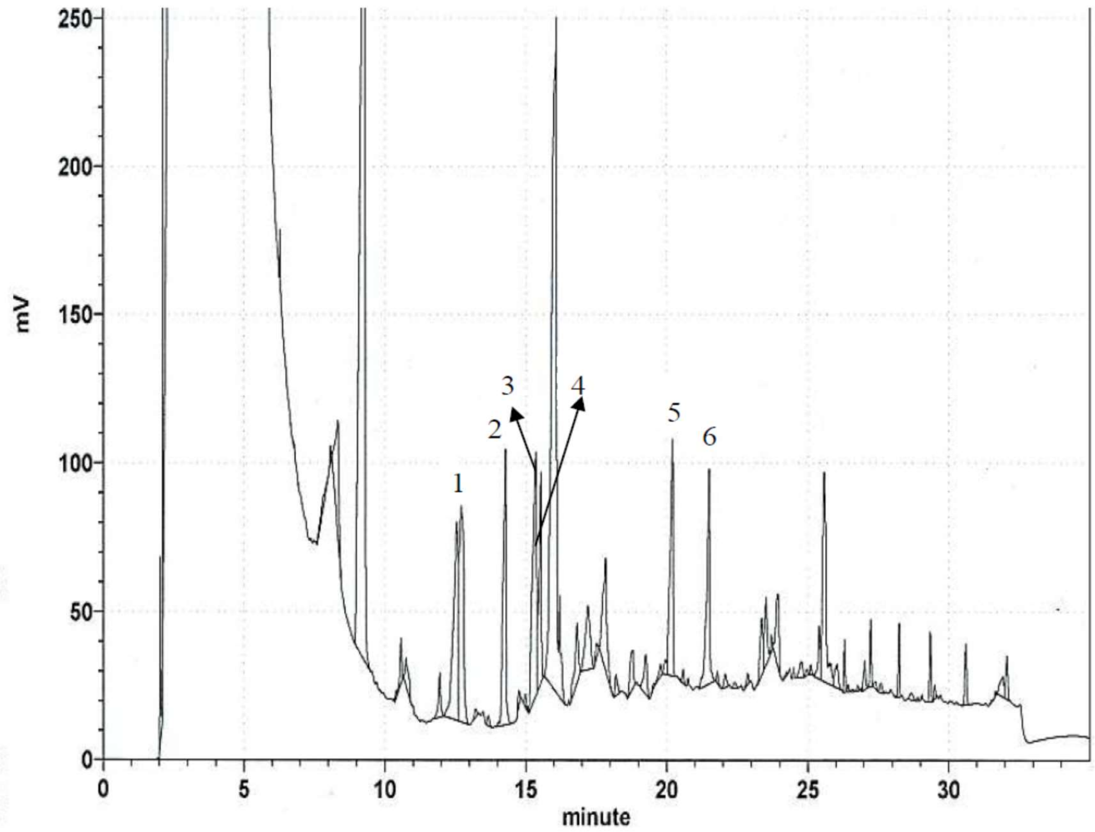

Peak assignment: 1: Glycine; 2: Valine; 3: Leucine; 4: Isoleucine; 5: Phenylalanine; 6: Cysteine.

Fig. S3b) GC CF-2

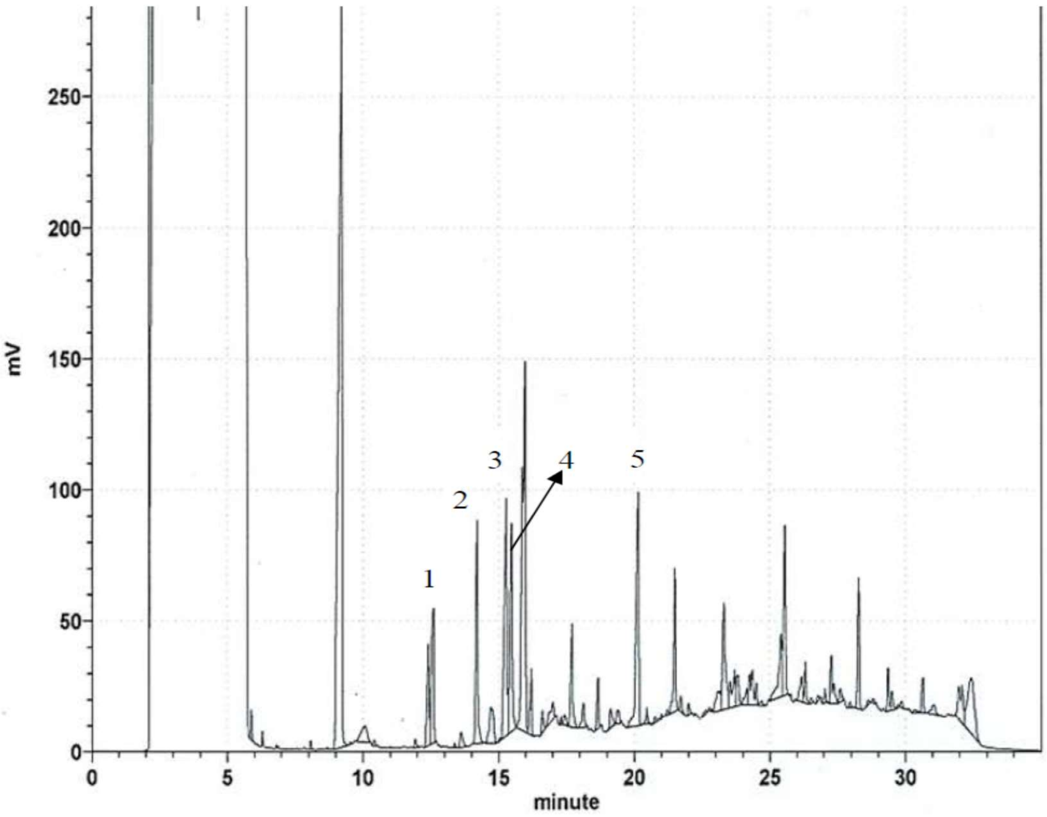

Peak assignment: 1: Glycine; 2: Valine; 3: Leucine; 4: Isoleucine; 5: Phenylalanine

Fig. S3c) GC CF-3 GC

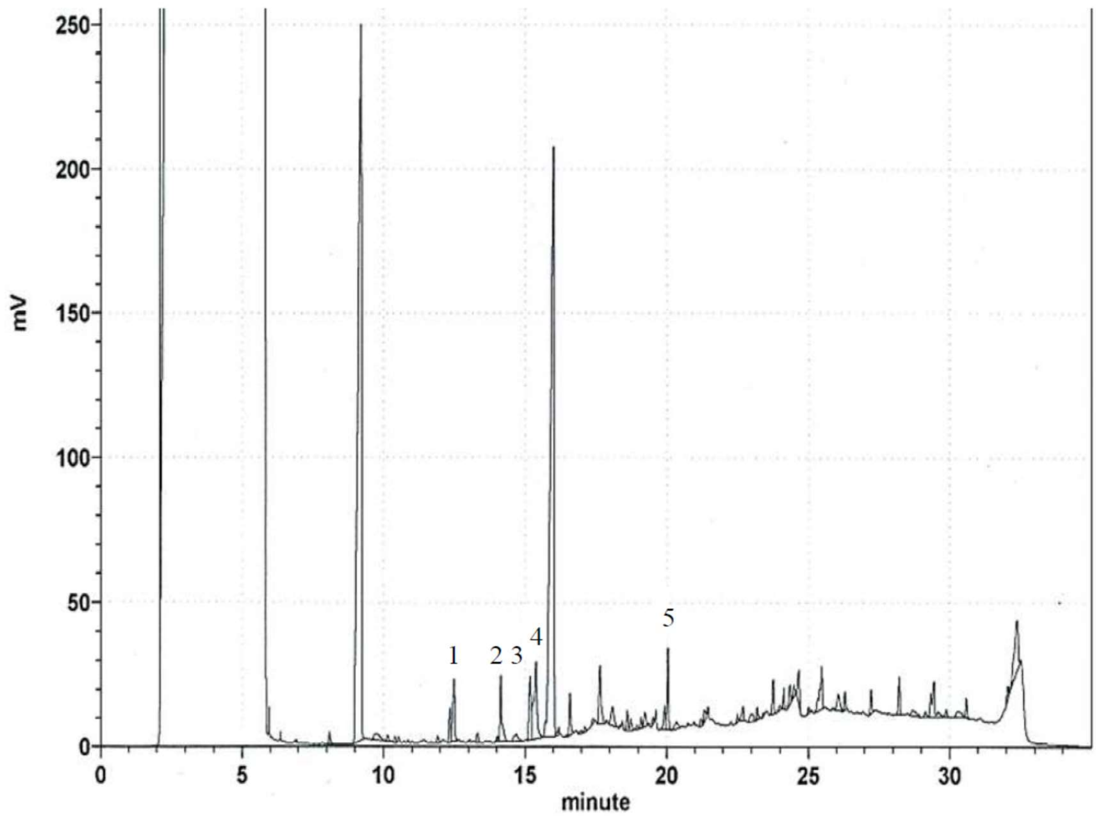

Peak assignment: 1: Glycine; 2: Valine; 3: Leucine; 4: Isoleucine; 5: Phenylalanine

Fig. S3d) GC CF-4 GC

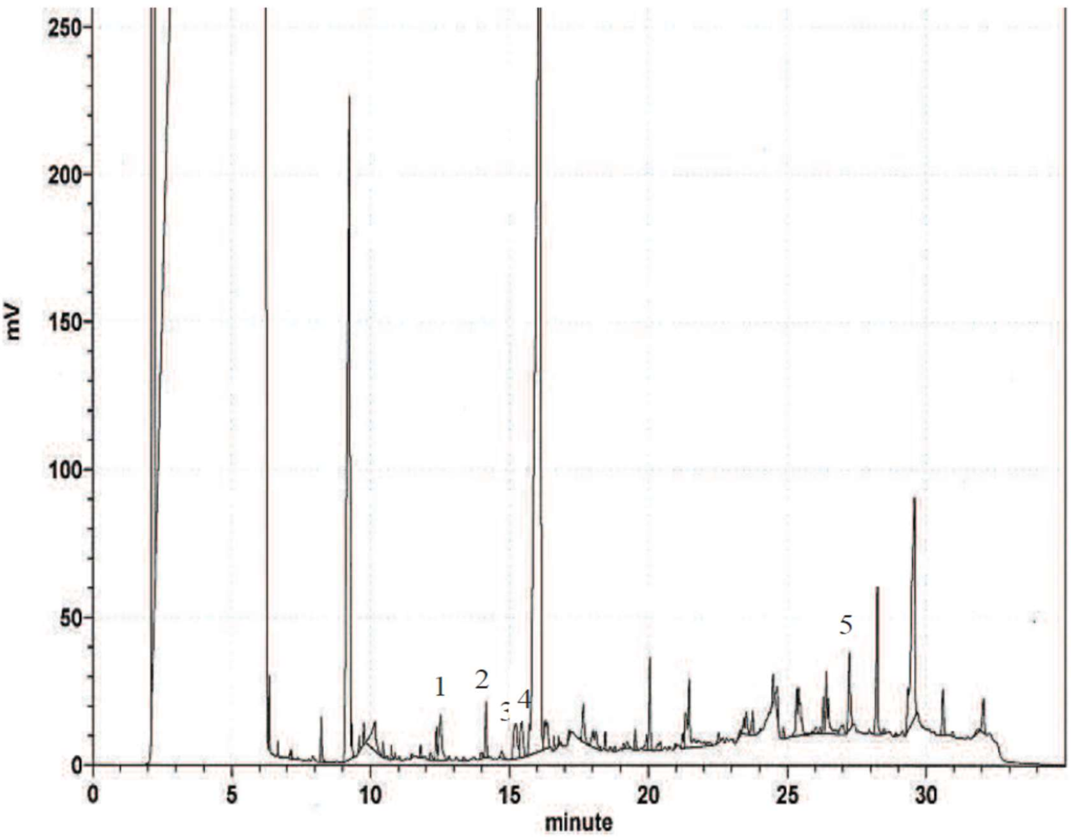

Peak assignment: 1: Glycine; 2: Valine; 3: Leucine; 4: Isoleucine; 5: Tyrosine
