# Supplementary material for: Nutrient composition of Chenopodium formosanum Koidz. bran: Fractionation and bioactivity of its soluble active polysaccharides
Source: PeerJ. 2022 May 25;10:e13459. doi: 10.7717/peerj.13459 (PMC9147384; doi:10.7717/peerj.13459)
Supplement: Supplemental Information 9 [file peerj-10-13459-s009.docx]

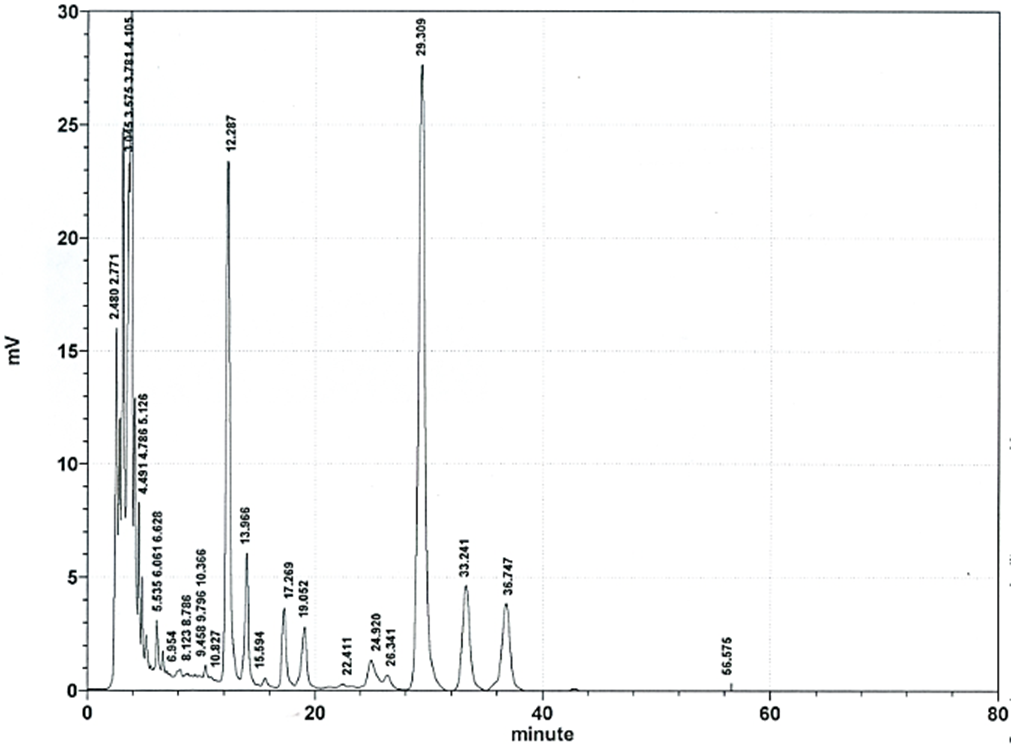


HPLC analysis for the monosaccharide composition present in the polysaccharide fraction CF-1 isolated from the hulls of C. formosanum Koidz. CF-1: the 3-fold ethanol precipitate from the hot water extracts.


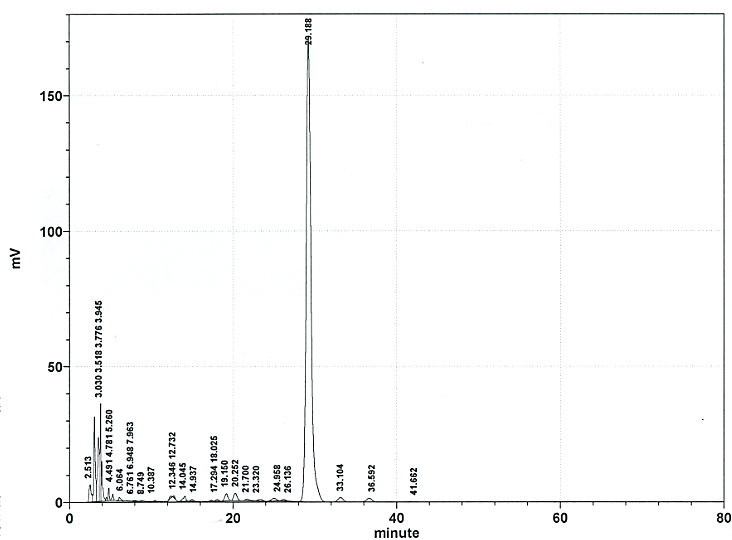


HPLC analysis for the monosaccharide composition present in the CF-2 polysaccharide fraction isolated from the hulls of C. formosanum Koidz. CF-2: the isoelectric precipitate from the 2%-NaOH extracts.


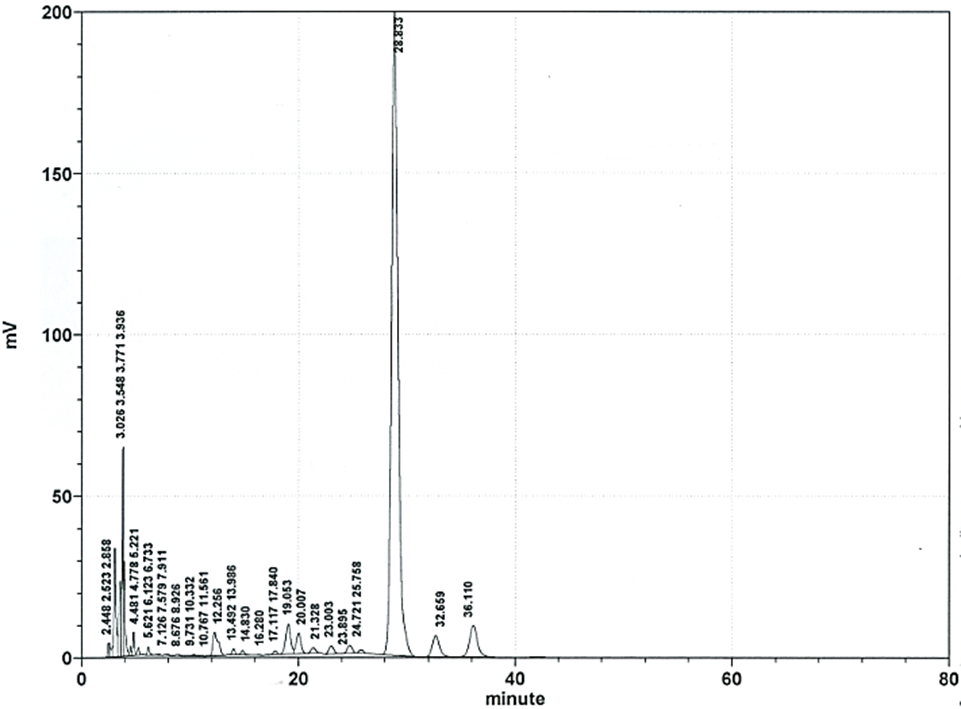


HPLC analysis for the monosaccharide composition present in the polysaccharide fraction CF-3 isolated from the hulls of *C. formosanum* Koidz.CF-3: the 3-fold ethanol precipitate from the 2%-NaOH extracts post isoelectric precipitation.


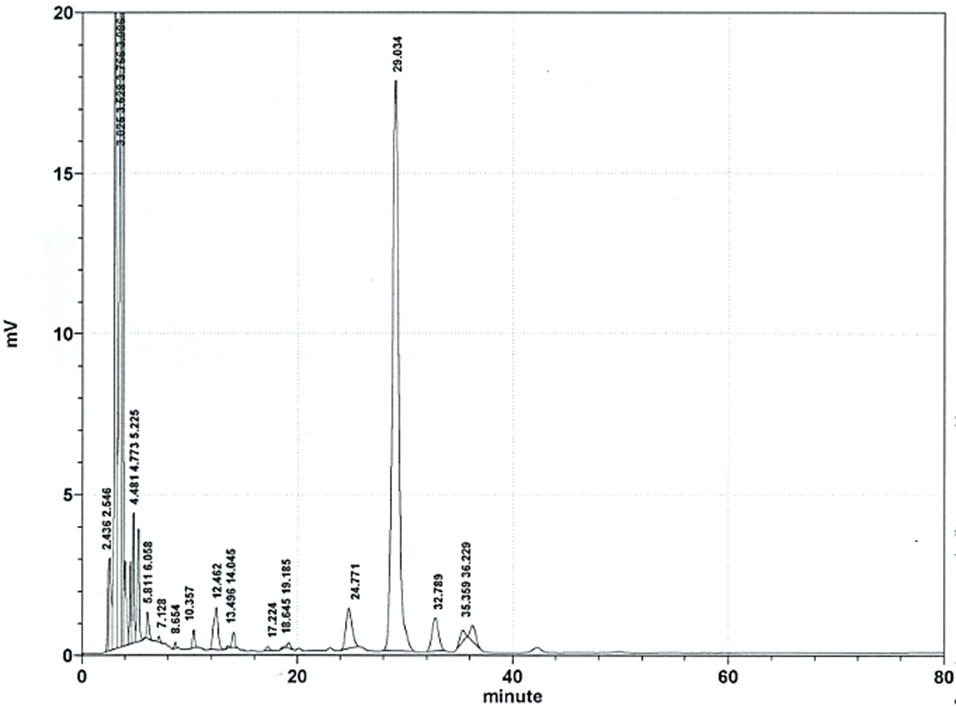


HPLC analysis for the monosaccharide composition present in the polysaccharide fraction CF-4 isolated from the hulls of C. formosanum Koidz.CF-4: the 3-fold ethanol precipitate from the 10%-KOH extracts post isoelectric precipitation.
